# Comparison of lithium levels between suicide and non-suicide fatalities: Cross-sectional study

**DOI:** 10.1038/s41398-022-02238-9

**Published:** 2022-11-07

**Authors:** Shuntaro Ando, Hideto Suzuki, Takehisa Matsukawa, Satoshi Usami, Hisanori Muramatsu, Tatsushige Fukunaga, Kazuhito Yokoyama, Yuji Okazaki, Atsushi Nishida

**Affiliations:** 1 Research Center for Social Science and Medicine, Tokyo Metropolitan Institute of Medical Science, 2-1-6 Kamikitazawa, Setagaya-ku, Tokyo, 156-8506 Japan; 2 Department of Neuropsychiatry, The University of Tokyo, 7-3-1 Hongo, Bunkyo-ku, Tokyo, 113-8655 Japan; 3 Tokyo Medical Examiner’s Office, 4-21-18 Otsuka, Bunkyo-ku, Tokyo, 112-0012 Japan; 4 Department of Forensic Medicine, Jichi Medical University, 3311-1 Yakushiji, Shimotsuke, Tochigi, 329-0498 Japan; 5 Department of Epidemiology and Environmental Health, Juntendo University, 2-1-1 Hongo, Bunkyo-ku, Tokyo, 113-8421 Japan; 6 Graduate school of Education, The University of Tokyo, 7-3-1 Hongo, Bunkyo-ku, Tokyo, 113-0033 Japan; 7 National Research Institute of Police Science, 6-3-1 Kashiwanoha, Kashiwa-shi, Chiba, 277-0882 Japan; 8 Michinoo Hospital, 1-1 Nijigaoka, Nagasaki-shi, Nagasaki, 852-8055 Japan

**Keywords:** Predictive markers, Molecular neuroscience

## Abstract

Ecological studies have suggested the protective effect of micro-dose lithium in drinking water against suicide, however, the association between body lithium level and suicide is unknown. We aimed to compare body lithium levels between suicide and non-suicide fatalities. This cross-sectional study included 12 suicides and 16 non-suicides who were examined or dissected at the Tokyo Medical Examiner’s Office from March 2018 to June 2021. The aqueous humor lithium concentration was measured twice using inductively coupled plasma mass spectrometry. Analysis of covariance (ANCOVA) was used to compare the lithium concentration between suicides and non-suicides. Mixed-effects model was conducted to account for all lithium concentration data. The aqueous humor lithium concentration did not change after death (*t*(7) = −0.70, $\bar v = - 0.02$, SE = 0.03, 95% CI = [−0.09, 0.05], *P* = 0.51, Cohen’s *d* = 0.01). The aqueous humor lithium concentration was lower in suicides (mean 0.50 μg/L (variance *s*^*2*^ 0.04)) than in non-suicides (mean 0.92 μg/L (*s*^*2*^ 0.07)) (*t*(26) = 4.47, $\bar v = 0.42$, SE = 0.09, 95% CI = [0.22 to 0.61], *P* < 0.001, Cohen’s *d* = 1.71). The ANCOVA showed that death by suicide was significantly associated with lower lithium concentration (*F*(1, 24) = 8.57, *P* = 0.007), and the effect size was large (*η*_*p*_^*2*^ = 0.26). The random intercept model showed a significant effect of suicide on aqueous humor lithium concentration (*b* = −0.261, SE = 0.102, 95% CI = [−0.471 to −0.051], *t*(24) = −2.568, *P* = 0.017). The results of this study demonstrate that even micro-dose lithium is associated with suicide death. Clinical studies are warranted to examine the effects of micro-dose lithium on suicide prevention.

## Introduction

More than 700,000 people die by suicide every year globally, and suicide is the fourth leading cause of death among individuals aged 15–29 years [1]. Although the age-standardized mortality rate for suicide has decreased since 1990, the suicide rate has been increasing [2]. Approximately 90% of suicides are due to mental disorder [3], but many people do not receive any mental health treatment from professionals at the time of suicide [4]. Although several strategies are effective for preventing suicide [5], further preventive measures are required.

Suicide is a multifactorial event caused by a complex interaction between biological, psychological, and environmental factors [6]. Although its biological basis has been uncertain, dysregulation in stress response systems, particularly the hypothalamic-pituitary-adrenal axis, was speculated as a diathesis for suicide [7]. Such dysregulation may affect downstream neuroinflammation, glutamatergic function, and neuronal plasticity [7]. Few drugs, including lithium, antidepressant, clozapine, and ketamine, have been suggested as protective against suicide [8]. Among them, lithium has the most robust evidence. Lithium carbonate was introduced for manic depressive illness in 1949 [9], and a meta-analysis of randomized controlled trials (RCTs) for mood disorders showed lithium’s protective effect against suicide [10]. Lithium may have a suicide prevention effect on aggression and impulsivity [11, 12], but it has been underutilized possibly due to commercial bias, narrow therapeutic range, and adverse effects [13, 14].

Epidemiological studies have also suggested the protective effect of micro-dose lithium against suicide. Lithium is mobilized by weathering processes and is transported into soils, from which it is taken up by plants and enters the food chain [9]. Lithium is absorbed from the human intestinal tract, and it is primarily excreted via the kidneys [9]. A meta-analysis of ecological studies showed that higher lithium concentration in drinking water is associated with reduced regional suicide rates [15, 16]. However, a publication bias was indicated in the meta-analysis [15], and ecological fallacy should be considered for population-level studies. Few individual-level studies have reached no definitive conclusion about the association between micro-dose lithium and suicidal behaviors. A semi-individual-level study showed that lithium concentration in drinking water is associated with depressive symptoms but not with suicidal ideation in general adolescents [17]. An individual-level study on adult patients who were transferred to an emergency department reported that serum lithium level is lower in suicide attempters than in controls [18, 19]. Further, a case study suggested that the lithium concentration in the brain of suicides is slightly lower than that in non-suicides [20], and another study revealed that lithium concentration ratio of white matter to gray matter in the brain was different between suicide victims and non-suicide cases [21, 22].

However, it has been unclear whether micro-dose lithium level in body was associated with suicide death or not. Therefore, the aim of this study was to investigate the difference in body lithium levels in these groups. We hypothesized that the body lithium levels were lower in suicides than in non-suicides.

## Materials and methods

### Study design, settings, and participants

This cross-sectional study compared the lithium concentrations in the aqueous humor of suicides and non-suicides. The subjects included 29 people who were examined or dissected at the Tokyo Medical Examiner’s Office from March 2018 to June 2021. All sudden unexpected deaths in the special wards of metropolitan Tokyo (9.64 million residents, 627.6 km^2^), including those reported as sudden unexpected deaths from disease, non-disease-related cause (e.g., suicide), or unknown causes, are reported to the Tokyo Medical Examiner’s Office. Medical examiners perform postmortem examinations to determine the manner and cause of death under Article 8 of the Corpse Autopsy and Preservation Law. The manner of death (suicide) was determined by the police investigation (e.g., past suicidal attempts, farewell note). We excluded cases of accidental death from suicidal cases. Information about medication was obtained by police. This study was approved by the ethics committee of the Tokyo Metropolitan Institute of Medical Science (approval number: 16-45), Tokyo Medical Examiner’s Office (29–2), and Juntendo University (approval number: 18-009). Informed consent was obtained from relatives within the third degree of relationship through an opt-out methodology on a website. Subjects whose relatives refused study participation were excluded. Cases in which post-mortem changes progressed (the late corpse phenomenon occurred) were also excluded from the study.

### Measurement of lithium concentration in the aqueous humor

Lithium is uniformly distributed in body water, with only a small difference between the extracellular and intracellular levels [9]. Postmortem assessment of electrolyte levels is often difficult, as levels may be significantly affected by the types of fluids, postmortem interval, analytical methods, and the amount of fluid [23]. Anatomically isolated ocular fluids (i.e., aqueous humor, vitreous humor) are presumed to be more available for postmortem biochemistry analysis because of fewer postmortem influences [23, 24]. A previous study reported that the metabolomic changes in ocular fluids are slower and smoother than in blood [25]. We inserted 26G needle into the anterior chamber, and collected aqueous humor by using 1 mL syringe during the examination and dissection. We did not record the amount of aqueous humor we took, however, we could take 100–200 μL from unilateral eye of the cases. Then the samples were immediately stored at 4 °C. The lithium concentration of the sample was then measured using inductively coupled plasma mass spectrometry (ICP-MS Agilent 8800; Agilent Technologies, California, USA) at Juntendo University. Ocular aqueous humor is a liquid, but it may contain organic matter. Therefore, we carefully performed pretreatment to digest the organic matter in order to reduce the matrix effect of ICP-MS analysis. Each 40 µL aqueous humor was placed into a perfluoro alkoxy Teflon vial (GL Sciences Inc., Tokyo, Japan) and digested with 80 µL of concentrated nitric acid (Ultrapure Grade, Tama Chemicals Co., Kawasaki, Japan), 50 µL hydrogen peroxide (Ultrapure Grade, Tama Chemicals Co.), and 330 µL ultrapure water in a microwave oven (MLS-1200 MEGA, Milestone S.R.L., Bergamo, Italy) in the following five steps, the power was set at 250, 0, 250, 400, and 600 W for 5, 1, 5, 5, and 5 min, respectively. The volume of the digested sample was then adjusted to 500 µL with ultrapure water. The 150 µL digested solution was diluted 2 times with 0.5% HNO_3_ and 200 μg/L beryllium as an internal standard (dilution factor was 25 for each sample). Prior to measurement, the lithium concentration of the certified standard substance (BCR 304, European Commission Joint Research Center Institute for Reference Materials and Measurements, Geel, Belgium) was determined, and the error was confirmed within the allowable range. The detection limit of lithium was 0.14 μg/L. For accuracy, the lithium concentration in the aqueous humor was measured twice (measurement 1 and 2 [M1 and M2]), and the average value of the two measurements was used for the analysis. According to a previous study [18], an expected effect size (standardized mean difference) was set at *d* = 1.0. Power analysis showed that the statistical power was approximately 0.8 when *d* = 1.0 and *n* = 15; thus, we aimed to gather 15 subjects for each group.

### Statistical analysis

Statistical analyses were conducted by a biostatistician (SU) who was blinded to the sample allocation and the study hypothesis using R (version 4.0.3) and SPSS (version 22, USA).

#### Correlation of lithium concentration in the aqueous humor and serum

Serum was also collected from the bodies of 17 people to verify whether the lithium concentration in the aqueous humor correlated with that in the serum. The lithium concentration in the serum was measured using the same method used for the aqueous humor.

#### Examination of post-mortem changes

The aqueous humor was collected twice every 16 h from the bodies of eight people to verify whether the lithium concentration in the aqueous humor did not change after death. The concentration of the first lithium (time 1 [T1]) collected in the aqueous humor of the eight people was compared with that in the aqueous humor collected 16 h later (time 2 [T2]).

#### Comparison of lithium concentration in the aqueous humor of suicides and non-suicides

We conducted analysis of covariance (ANCOVA) to compare the lithium concentration in the aqueous humor between suicides and non-suicides. Age and sex were treated as a priori confounders in the analyses. Additional analyses were conducted using M1 or M2 data instead of the mean value of the two measurements (M1 and M2).

Further analysis using mixed-effects model was conducted to confirm how major results about the effect of suicide can change if we account for all observations of M1 and M2 aqueous humor lithium concentration data at T1 and T2 and model each observation directly (rather than using mean values). Postmortem interval (time or level-1 variable) was included as a covariate in this analysis, and both random intercept model and random intercept and random slope model were specified to evaluate the effect of suicide (as individual or level-2 variable). Restricted maximum likelihood was used to estimate parameters in this analysis.

Since many of the suicides died by hanging, the lithium concentration in the aqueous humor was compared between individuals who died by hanging and those who died by other suicide methods.

### Patient and public involvement

No patients or members of the public were involved in the design, conduct, reporting, or dissemination plans of our research.

## Results

Blood and/or aqueous humor were collected from the bodies of 29 people (13 suicides and 16 non-suicides, 20 males and 9 females). Lithium was not taken as a medication by any subjects. Lithium concentration in the aqueous humor of 28 samples could be measured, except for one whose aqueous humor volume was small. The mean age was 40.4 years (SD (standard deviation) 17.6) in suicides and 61.4 years (SD 14.0) in non-suicides (Table 1). The female ratio was 44% in suicides and 56% in non-suicides. The measurement error of the reference material was within 5.7% of the certified value. The methods of suicide were hanging (9 people), acute drug intoxication (2), oxygen-deficient asphyxiation (1), and multiple injury (1). The causes of death in non-suicides include ketoacidosis (3), drowning (3), myocardial infarction (1), pulmonary artery embolus (1), subdural hematoma (1), and undernutrition (1). The paired *t*-test showed no significant difference (denoted as $\bar v$) between the two measurements (M1 and M2) of lithium concentration in the aqueous humor (*t*(27) = −1.56, $\bar v = 0.04$, SE = 0.03, 95% CI [confidence interval] = [−0.01, 0.09], *P* = 0.13, Cohen’s *d* = 0.01) (Table S1).

**Table 1:** Demographic characteristics of the suicides and non-suicides.

	Suicides (*n* = 13)	Non-suicides (*n* = 16)	*p* Value
Age, mean (SD), years	40.4 (17.6)	61.4 (14.0)	0.061
Sex, No. (%), female	4 (44.4)	5 (55.6)	0.98
Postmortem interval, mean (SD), hours	20.6 (9.4)	49.8 (38.2)	0.003

### Correlation of lithium concentration in the aqueous humor and serum

The Shapiro–Wilk test suggested that the lithium concentrations in the aqueous humor and the serum were normally distributed (*W* = 0.96, *P* = 0.43, and *W* = 0.93, *P* = 0.25) (Table S2). The lithium concentrations in the aqueous humor and serum were significantly correlated (Pearson’s correlation coefficient (*r*) = 0.92, 95% CI = [0.77, 0.97], *t*(14) = 8.61, *P* < 0.001).

### Examination of post-mortem changes

The Shapiro–Wilk test suggested that the lithium concentration in the aqueous humor was normally distributed both in the first (T1) and second (T2) data (W = 0.91, *P* = 0.36, and *W* = 0.90, *P* = 0.27) (Table 2). The paired *t*-test showed no significant difference between T1 and T2 data (*t*(7) = −0.70, $\bar v = - 0.02$, SE = 0.03, 95% CI = [−0.09, 0.05], *P* = 0.51, Cohen’s *d* = 0.01).

**Table 2:** Examination of post-mortem change: two measurements of aqueous humor lithium levels 16 h apart.

Case	Time 1 (μg/L)	Time 2 (μg/L)
3	0.78	0.95
4	0.82	0.74
5	1.02	0.94
6	0.87	0.85
7	0.70	0.73
8	0.78	0.85
9	0.71	0.73
10	0.98	1.02

### Comparison of lithium concentration in the aqueous humor of suicides and non-suicides

**Fig. 1:**
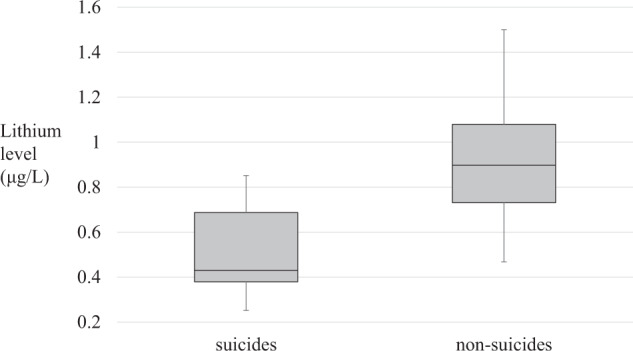
Aqueous humor lithium levels in suicides and non-suicides.

Shapiro–Wilk test suggested that the lithium concentration in the aqueous humor was normally distributed in suicides and non-suicides (*W* = 0.90, *P* = 0.15, and *W* = 0.97, *P* = 0.87). The *t*-test showed that the mean concentration in the aqueous humor was significantly lower in suicides (0.50 μg/L (variance (*s*^*2*^) = 0.04)) than in non-suicides (0.92 μg/L (*s*^*2*^ = 0.07)), with showing large effect size of Cohen’s *d* (*t*(26) = 4.47, $\bar v = 0.42$, SE = 0.09, 95% CI = [0.22,0.61], *P* < 0.001, Cohen’s *d* = 1.71) (Fig. 1). The ANCOVA was performed with lithium concentration in the aqueous humor as the dependent variable, suicide as a fixed factor, and age and sex as covariates. The ANCOVA showed a significant relationship between suicide and lithium concentration in the aqueous humor, even after controlling for age and sex (*F*(1, 24) = 8.57, *P* = 0.007), and the effect size was large (*η*_*p*_^*2*^ = 0.26) (Table 3). As supplemental information, the partial correlation between suicide and lithium concentration in the aqueous humor was calculated as 0.51 (95% CI = [0.17, 0.74], *P* = 0.007). These results were similar regardless of the choice of lithium concentration measurements (M1 and M2) used for the analysis.

**Table 3:** Estimated result of ANCOVA comparing aqueous humor lithium levels in suicides and non-suicides.

	Unadjusted model					Adjusted model				
	*B*	SE *B*	*β*	*t*	*P*-value	*B*	SE *B*	*β*	*t*	*P*-value
Suicide	−0.42	0.09	−0.66	−4.47	<0.001	−0.31	0.11	−0.49	−2.93	0.007
Age						0.01	0.00	0.28	1.68	0.11
Sex (male)						−0.15	0.10	−0.22	−1.53	0.14
	*R*^*2*^ = 0.41					*R*^*2*^ = 0.47				

The additional analysis using random and slope model showed the significant effect of suicide on aqueous humor lithium concentration (*b* = −0.344, SE = 0.089, 95% CI = [−0.528, −0.161], t(26) = −3.855, *P* = 0.001), but not for postmortem interval (*b* = 0.0022, SE = 0.0014, 95%CI = [−0.0005, 0.0050], *t*(70) = 1.658, *P* = 0.10). When age and sex were included as confounders, only the random intercept model was identified and it showed the significant effects of suicide (*b* = −0.261, SE = 0.102, 95% CI = [−0.471 to −0.051], *t*(24) = −2.568, *P* = 0.017) as well as postmortem interval (*b* = 0.0025, SE = 0.0012, 95%CI = [0.00002 to 0.00489], *t*(70) = 2.011, *P* = 0.048) on aqueous humor lithium concentration. Among the suicides, the lithium level in the aqueous humor was not significantly different between individuals who died by hanging and those who died by other methods (*t*(10) = 0.42, $\bar v = 0.05$, SE = 0.13, 95% CI = [−0.23,0.34], *P* = 0.68, Cohen’s *d* = 0.02).

## Discussion

The main new finding of this study is the lower aqueous humor lithium level in suicides than in non-suicides. We collected aqueous humor, which is less susceptible to post-mortem changes, and measured the lithium concentration with high accuracy. As expected, the lithium concentration in the aqueous humor reflected that in the serum and did not significantly change after death. The findings of this study could lead to deeper understanding of the role of micro-dose lithium in suicide.

The lithium concentration in the aqueous humor was lower in suicides than in non-suicides. As expected, the lithium concentration in the aqueous humor reflected that in the serum and did not significantly change after death. This result is consistent with previous ecological studies showing an inverse correlation between regional lithium concentration in drinking water and suicide rate [15]. The findings of the present study also accord with those of a previous study, suggesting that the lithium concentration in the brain is lower in suicide than in a non-suicide victim [20]. The lithium concentration observed in this study was as small as [20] or even smaller than that in previous studies [18]. Since the lithium concentration in this study was less than 1/1000 of the clinically effective concentration (0.4–1.2 mEq/L), we could assume that even micro-dose lithium may be associated with suicidality.

Although the biochemical action of lithium is complex, enhancement of the transport of vitamin B12 and folate, which affect mood-associated parameters, may be a mechanism of the antidepressive, mood-elevating, and antiaggressive actions of lithium at nutritional dosage [9]. Several studies showed the beneficial neuropsychiatric effects of the nutritional dosage of lithium corresponding to approximately 1/1000 of clinical dosage. An RCT has revealed that 400 µg of lithium daily for 4 weeks improves mood and stability more than placebo in former substance abusers [26]. Another RCT showed that 300 µg of lithium daily for 15 months prevents cognitive loss compared with placebo in patients with Alzheimer’s disease [27]. In therapeutic dose, several neurodevelopmental effects of lithium, including increase in brain-derived neurotrophic factor [28], reduction in glial cell line-derived neurotrophic factor concentration in the cerebrospinal fluid [29], decrease in phosphorylated tau in cerebrospinal fluid [30], and inhibition of glycogen synthase kinase-3, were reported [31]. Furthermore, a therapeutic dose of lithium may have morphological effects on the brain including increased volume of white matter, dorsolateral prefrontal cortex, anterior cingulate gray matter [32], and hippocampus [33].

Several explanations may be inferred for the lower lithium concentration in suicide victims than in non-suicide victims. First, suicide victims might be depressive and have lost appetite, thus having lower dietary lithium intakes than non-suicide victims. Humans take lithium from grains and vegetables (0.5–3.4 mg/kg), daily products (0.5 mg/kg), and meat (0.012 mg/kg), and the estimated daily lithium intakes range from 348 to 1560 µg depending on the country [9]. Since 14.3 µg/kg body weight lithium intake is recommended [9], individuals with low appetite could become lithium deficient. Second, suicide victims might have different absorption and excretion properties of lithium compared with non-suicide victims. For example, patients with kidney disease have lower lithium concentration than normal [9]. In either way, the suicide victims might be lithium deficient. While lithium deficiency causes behavioral changes, such as diminished conditioned avoidance behavior in animals [9, 34], that lithium deficiency was suggested to precipitate impulsivity in humans [35].

Several important clinical implications can be inferred from the finding of this study. Since we did not limit the study samples in any psychiatric diagnosis, this study findings may mean that micro-dose lithium has an important role in suicide regardless of the psychiatric diagnosis. The possibility of micro-dose lithium as a potential candidate for the treatment of suicidality should be considered for patients regardless of medical diagnosis. Further, the findings of our study suggested the possibility that body lithium level could be a biological marker for suicidality. Future longitudinal observational study is warranted to investigate the property of body lithium level as a biological marker in predicting suicidality. Although not shown in the results due to small number of cases (*n* = 17) and only once measurement, the serum lithium level was lower in suicides (mean 0.46 μg/L, SD 0.25) than in non-suicides (mean 1.03 μg/L, SD 0.28) in this study. This lithium level in non-suicides seemed to be lower than the controls in the previous study [19], and the difference may be due to post-mortem change of serum which we did not treat in this study. The difference of lithium levels between suicide attempters and completers would be a future research focus.

This study has several strengths and limitations. The study site covered a wide area of Tokyo with more than nine million residents. Lithium measurement was conducted twice with highly accurate method. The major potential limitation of our study is the generalizability of the findings. Although this study covered a wide area, the study sample was collected in only one city. Thus, the association observed in this study could not be generalized to residents in other cities or countries. Since the number of cases was small and only a few adolescents were included in this study, the generalizability of the finding to adolescents would be questionable. We could not exclude the effect of other nutrients on suicide because of the absence of body weight and other nutritional element data.

Future studies including child and adolescent cases in different regions are warranted to replicate this study. In such replication study, body weight, kidney function, and other nutrients should be measured. The potential minimum effective dose of lithium in preventing suicide should be examined. As a previous study showed that 30 µg/L or more lithium concentration in drinking water is associated with lower suicide rate [36], a certain minimum dose of lithium may be required to achieve anti-suicidal properties. Although several studies have examined the effect of micro-dose lithium on mood or cognitive impairment [26, 27, 37], no clinical trial has investigated the effect of micro-dose lithium on suicide. Clinical trials are warranted to examine the effect of micro-dose lithium on suicide prevention.

## Conclusions

In conclusion, the results of this study showed that the lithium levels in the body were lower in suicides than in non-suicides, suggesting that even micro-dose lithium has an important role in suicidality. Important next steps will be clinical studies which examine the effects of micro-dose lithium on suicide prevention.

## Supplementary information

Two measurements of lithium levels in each aqueous humor sample (μg/L)

Demographic characteristics and lithium levels in the study cases
