# Correction: Comparison of lithium levels between suicide and non-suicide fatalities: cross-sectional study

**DOI:** 10.1038/s41398-023-02359-9

**Published:** 2023-02-13

**Authors:** Shuntaro Ando, Hideto Suzuki, Takehisa Matsukawa, Satoshi Usami, Hisanori Muramatsu, Tatsushige Fukunaga, Kazuhito Yokoyama, Yuji Okazaki, Atsushi Nishida

**Affiliations:** 1 Research Center for Social Science and Medicine, Tokyo Metropolitan Institute of Medical Science, 2-1-6 Kamikitazawa, Setagaya-ku, Tokyo, 156-8506 Japan; 2 Department of Neuropsychiatry, The University of Tokyo, 7-3-1 Hongo, Bunkyo-ku, Tokyo, 113-8655 Japan; 3 Tokyo Medical Examiner’s Office, 4-21-18 Otsuka, Bunkyo-ku, Tokyo, 112-0012 Japan; 4 Department of Forensic Medicine, Jichi Medical University, 3311-1 Yakushiji, Shimotsuke, Tochigi, 329-0498 Japan; 5 Department of Epidemiology and Environmental Health, Juntendo University, 2-1-1 Hongo, Bunkyo-ku, Tokyo, 113-8421 Japan; 6 Graduate school of Education, The University of Tokyo, 7-3-1 Hongo, Bunkyo-ku, Tokyo, 113-0033 Japan; 7 National Research Institute of Police Science, 6-3-1 Kashiwanoha, Kashiwa-shi, Chiba, 277-0882 Japan; 8 Michinoo Hospital, 1-1 Nijigaoka, Nagasaki-shi, Nagasaki, 852-8055 Japan

**Keywords:** Predictive markers, Molecular neuroscience

Correction to: *Translational Psychiatry* 10.1038/s41398-022-02238-9, published online 07 November 2022

The original version of this article contained an error in the abstract. The typo in the current paper is:

The aqueous humor lithium concentration was lower in suicides (mean 0.50 μg/L (variance s2 0.04)) than in non-suicides (mean 0.92 μg/L (s2 0.07)) (t(26) = 4.47, [Formula: see text], SE = 0.09, 95% CI = [0.22 to 0.61], *P* < 0.001, Cohen’s d = 1.71). The correct version should be:The aqueous humor lithium concentration was lower in suicides (mean 0.50 μg/L (variance s2 0.04)) than in non-suicides (mean 0.92 μg/L (s2 0.07)) (t(26) = 4.47, [Formula: see text], SE = 0.09, 95% CI = [0.22 to 0.61], *P* < 0.001, Cohen’s d = 1.71). The original article has been corrected.

