# Supplementary material for: Comparison of lithium levels between suicide and non-suicide fatalities: Cross-sectional study
Source: Transl Psychiatry. 2022 Nov 7;12:466. doi: 10.1038/s41398-022-02238-9 (PMC9640730; doi:10.1038/s41398-022-02238-9)
Supplement: Supplementary file 1 — Two measurements of lithium levels in each aqueous humor sample (μg/L) [file 41398_2022_2238_MOESM1_ESM.docx]

| Table S1. Two measurements of lithium levels in each aqueous humor sample (μg/L) | | | |
| --- | --- | --- | --- |
| Case | Measurement 1 | Measurement 2 | Mean |
| 1 | 1.41 | 1.60 | 1.50 |
| 2 | 1.10 | 1.02 | 1.06 |
| 3 | 1.01 | 0.89 | 0.95 |
| 4 | 0.80 | 0.69 | 0.74 |
| 5 | 0.98 | 0.90 | 0.94 |
| 6 | 0.82 | 0.89 | 0.85 |
| 7 | 0.76 | 0.70 | 0.73 |
| 8 | 0.86 | 0.85 | 0.85 |
| 9 | 0.74 | 0.72 | 0.73 |
| 10 | 1.04 | 1.00 | 1.02 |
| 11 | 0.73 | 0.62 | 0.67 |
| 12 | 0.73 | 0.84 | 0.78 |
| 13 | 0.38 | 0.33 | 0.35 |
| 14 | 0.71 | 0.76 | 0.73 |
| 15 | 0.39 | 0.40 | 0.40 |
| 16 | - | - | - |
| 17 | 0.18 | 0.32 | 0.25 |
| 18 | 0.43 | 0.49 | 0.46 |
| 19 | 0.33 | 0.45 | 0.39 |
| 20 | 0.35 | 0.45 | 0.40 |
| 21 | 0.28 | 0.29 | 0.28 |
| 22 | 0.46 | 0.50 | 0.48 |
| 23 | 0.86 | 0.71 | 0.79 |
| 24 | 1.32 | 1.17 | 1.24 |
| 25 | 0.86 | 0.59 | 0.72 |
| 26 | 1.20 | 1.25 | 1.22 |
| 27 | 1.27 | 1.00 | 1.14 |
| 28 | 0.56 | 0.38 | 0.47 |
| 29 | 0.77 | 0.42 | 0.60 |
