# Supplementary material for: Comparison of lithium levels between suicide and non-suicide fatalities: Cross-sectional study
Source: Transl Psychiatry. 2022 Nov 7;12:466. doi: 10.1038/s41398-022-02238-9 (PMC9640730; doi:10.1038/s41398-022-02238-9)
Supplement: Supplementary file 2 — Demographic characteristics and lithium levels in the study cases [file 41398_2022_2238_MOESM2_ESM.docx]

| Table S2. Demographic characteristics and lithium levels in the study cases | | | | | | |
| --- | --- | --- | --- | --- | --- | --- |
| Case | Sex | Age | Suicide | Postmortem interval (h) | Serum (μg/L) | Aqueous humor (μg/L) |
| 1 | M | 65 | No | 144 | - | 1.50 |
| 2 | F | 93 | No | 58.5 | 1.12 | 1.06 |
| 3 | M | 73 | No | 120 | - | 0.95 |
| 4 | M | 69 | No | 16 | - | 0.74 |
| 5 | M | 50 | No | 22 | - | 0.94 |
| 6 | M | 74 | Yes | 20 | - | 0.85 |
| 7 | M | 75 | No | 60 | 0.75 | 0.73 |
| 8 | M | 44 | No | 24 | 0.87 | 0.85 |
| 9 | F | 73 | No | 18 | - | 0.73 |
| 10 | F | 49 | No | 17 | - | 1.02 |
| 11 | F | 35 | Yes | 26 | 0.76 | 0.67 |
| 12 | F | 30 | Yes | 16 | 0.57 | 0.78 |
| 13 | M | 38 | Yes | 20 | 0.39 | 0.35 |
| 14 | M | 63 | Yes | 21 | 1.04 | 0.73 |
| 15 | M | 30 | Yes | 20 | 0.41 | 0.40 |
| 16 | F | 30 | Yes | 26 | 0.17 | - |
| 17 | F | 28 | Yes | 18 | 0.21 | 0.25 |
| 18 | M | 32 | Yes | 9 | 0.21 | 0.46 |
| 19 | M | 41 | Yes | 35 | 0.37 | 0.39 |
| 20 | M | 38 | Yes | 26 | 0.38 | 0.40 |
| 21 | M | 16 | Yes | 11 | 0.53 | 0.28 |
| 22 | M | 70 | Yes | 4 | 0.50 | 0.48 |
| 23 | M | 57 | No | 22 | 0.92 | 0.79 |
| 24 | F | 73 | No | 34 | 1.47 | 1.24 |
| 25 | M | 59 | No | 48 | - | 0.72 |
| 26 | M | 59 | No | 36 | - | 1.22 |
| 27 | F | 39 | No | 27 | - | 1.14 |
| 28 | M | 52 | No | 26 | - | 0.47 |
| 29 | M | 52 | No | 12 | - | 0.60 |
| M: male, F: female | | | | | | |
